# Tremulatory and Abdomen Vibration Signals Enable Communication through Air in the Stink Bug *Euschistus heros*

**DOI:** 10.1371/journal.pone.0056503

**Published:** 2013-02-27

**Authors:** Andreja Kavčič, Andrej Čokl, Raúl A. Laumann, Maria Carolina Blassioli-Moraes, Miguel Borges

**Affiliations:** 1 Department of Entomology, National Institute of Biology, Ljubljana, Slovenia; 2 Embrapa Genetic Resources and Biotechnology, Brasília, DF, Brazil; George Washington University, United States of America

## Abstract

Communication by substrate-borne mechanical signals is widespread among animals but remains one of their least understood communication channels. Past studies of vibrational communication in insects have been oriented predominantly to communication during mating, showing that species- and sex-specific vibrational signals enable recognition and localization of potential mates on continuous solid substrates. No special attention has been paid to vibrational signals with less obvious specificity as well as to the possibility of vibrational communication across substrates that are not in physical contact. We aimed to reinvestigate emission of the aforementioned vibrational signals transmitted through a plant in the stink bug *Euschistus heros* (Pentatomidae: Pentatominae) and to check whether individuals are able to communicate across adjecent, physically separated substrates. We used laser vibrometry for registration of substrate-borne vibrational signals on a bean plant. Using two bean plants separated for 3 to 7 cm between two most adjacent leaves, we investigated the possibility of transmission of these signals through air. Our study showed that males and females of *E. heros* communicate using tremulatory, percussion and buzzing signals in addition to the previously described signals produced by vibrations of the abdomen. Contrary to the latter, the first three signal types did not differ between sexes or between pentatomid species. Experiments with two physically separated plants showed significant searching behaviour and localization of vibrational signals of an *E. heros* male or a female, in response to abdominal vibration produced signals of a pair duetting on the neighbouring plant, in comparison to control where no animals were on the neighbouring plant. We also confirmed that transmission through air causes amplitude and frequency decay of vibrational signals, which suggests high-amplitude, low-frequency tremulatory signals of these stink bugs their most plausible way of communication across discontinuous substrates.

## Introduction

Substrate-borne sound communication is the most widely used insect way of information exchange with mechanical signals [1], [2]. Although their small body size prevents efficient emission of low frequency airborne sound [3], [4] Eriksson and co-authors [5] recently demonstrated airborne inter-plant communication with low frequency signals in the less than 5 mm body sized leafhopper *Scaphoideus titanus* Ball (Hemiptera: Cicadellidae). Despite of high attenuation during transmission through air, females standing on a leaf replied steadily to male calls emitted from another leaf separated by 6 cm air gap.

*Nezara viridula* L. (Hemiptera: Pentatomidae) and many other plant-dwelling species of the stink bug subfamily Pentatominae emit species-specific male pheromones with long range action [6], [7], [8] to attract mates on common places on plants. Once animals are closer together and on the same substrate, medium and short range signalling cues become prominent, namely substrate-borne vibrational signals, visual and tactile signals. Use of airborne component of substrate-borne vibrational signals could represent a potentially important suplementary mechanism for information exchange in mating behaviour [9], [10], [11] when use of other communications mechanisms is impossible or inefficient. This new communication channel could represent a shortcut not only for substrate-borne vibrational signals to reach the intended receiver, but also for the receiver to find the emitter of vibrational signals. What is more, airborne component of substrate-borne vibrational signals could enable communication between animals on substrates that are not in physical contact and communication by chemical, vibrational and visual signals is impossible or inefficient. The possibility for airborne sound communication remained completely ignored in Pentatomidae and other plant-dwelling Heteroptera, and the role of group characteristic vibrational signal frequency and velocity for efficient transmission through air has not been studied yet. Leafhoppers [5] and pentatominae stink bugs [12], [13] produce vibrational signals of similar velocity at the source but of different frequency characteristics: compared to leafhoppers’ vibrational signals, which contain also high frequency components (author’s personal communication), all until now investigated pentatominae stink bugs produce signals of lower frequencies tuned with the mechanical properties of plants [14], [15]. A broader repertoire of vibration producing mechanisms has been described in predatory stink bugs of the pentatomid subfamily Asopinae that emit high amplitude tremulatory signals [16], [17], [18], which have been ignored until now in Pentatominae. The role of different time parameters of pentatominae abdomen produced signals for mate recognition and location in the field is well understood [19]–[23] but communication distance with substrate- and/or airborne high amplitude and species less specific signals may be significantly increased.

The aim of the present study was to get answers to the following questions. (A) Do stink bugs of the subfamily Pentatominae produce vibrational signals also with mechanisms other than abdomen vibration? (B) If so, what are characteristics of such signals and in what behavioural context are they emitted? (C) Can pentatominae stink bugs communicate with airborne component of narrow-band low frequency signals produced by different mechanisms? (D) If so, what is the velocity and frequency relation between naturally emitted signals recorded simultaneously from plants of which parts are not in a contact. To get the answers to these questions we investigated in natural conditions, insects placed on bean plants, different aspects of vibrational communication in the stink bug species *Euschistus heros* whose abdomen vibration produced signals have been first described by Blassioli-Moraes and co-workers [24] from vibrations recorded on a non-resonant substrate.

## Results

**Figure 1:**
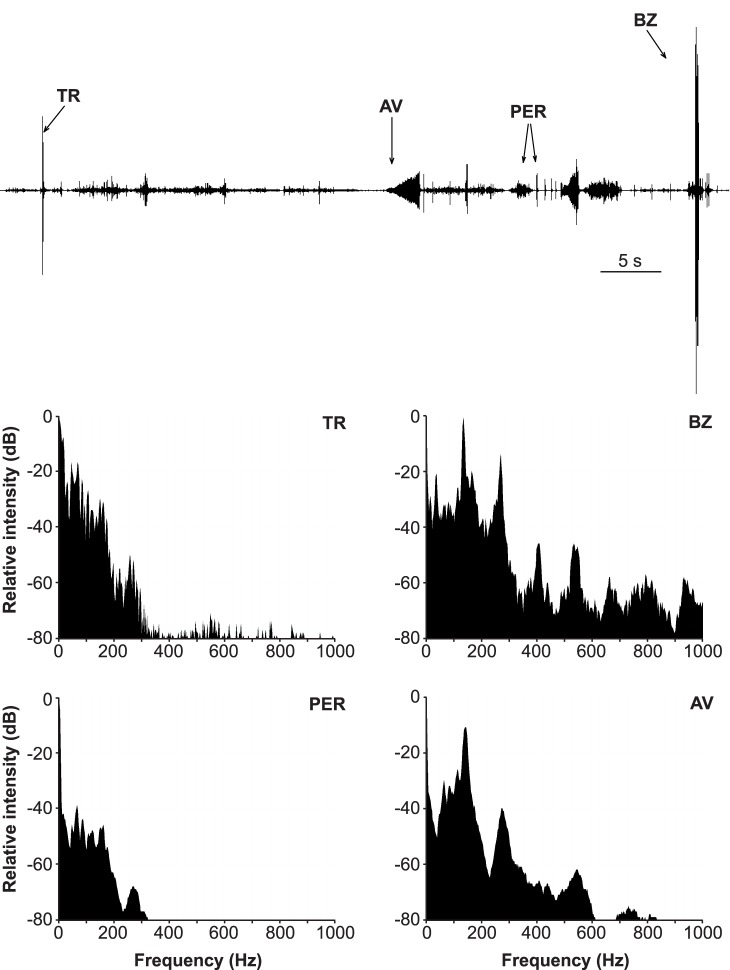
Spectral characteristics of tremulatory, buzzing, percussion and abdomen vibration produced signals. (Above) Vibrational signals produced by tremulation (TR), buzzing (BZ), percussion (PER) and by vibrating the abdomen (AV) on the leaf and recorded on the stem 14–16 cm from the source. Sequence shown is 1 minute long. (Below) Frequency spectra of single tremulatory, buzzing and abdomen vibration produced pulses, and of a 10 s sequence of percussion signals.

*E. heros* males and females emitted in our experimental conditions species-specific vibrational signals produced by abdomen vibration as described by Blassioli-Moraes and co-authors [24]. On the plant we recorded as first among Pentatominae also signals produced by tremulation (vigorous vibration of the whole body), percussion (tapping with forelegs on the ground) and buzzing (vibration of lifted wings) **(** **Figures 1** **,** **2** **;** **Table 1** **)**.

**Figure 2:**
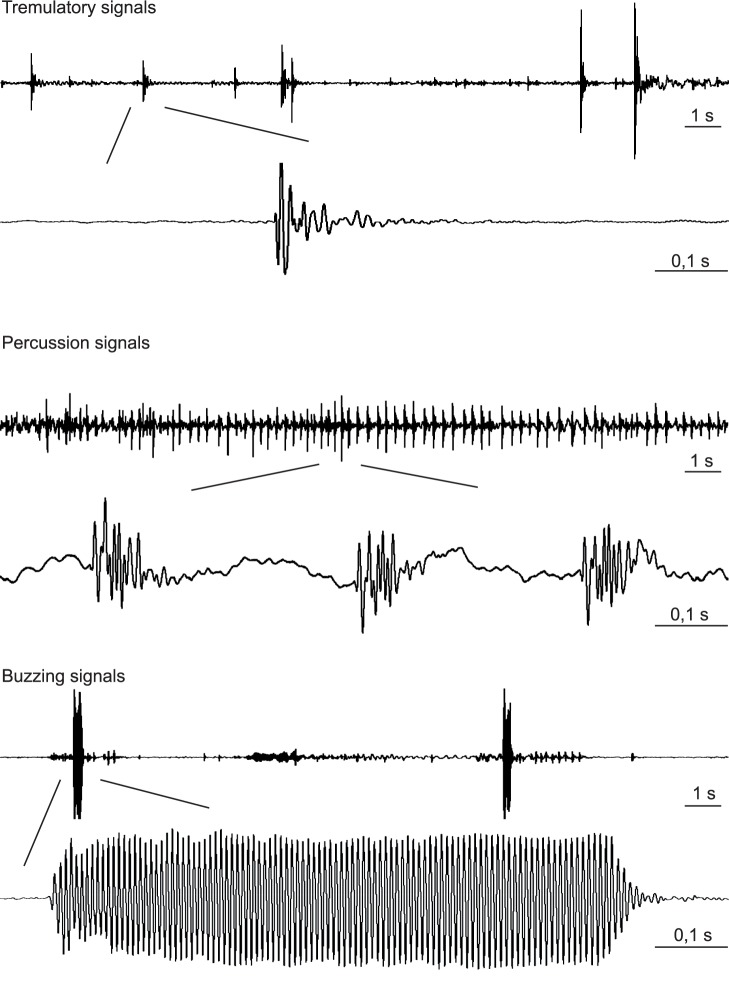
Oscillograms of tremulatory, percussion and buzzing signals. Signals were emitted by *E. heros* on the leaf of a bean plant and recorded on the stem 14–16 cm from the source.

**Table 1:** Parameters of vibrational signals produced by mechanisms other than abdomen vibration.

	DURATION (ms)	REPETITION TIME (ms)	VELOCITY (mm/s)
**Tremulatory signals**	300.4±160. 1 (n = 90, N = 3)	–	min. = 2.1±0.8 (n = 30) max. = 4.4±3.7 (n = 30)
**Percussion signals**	I: 128.4±20.8 (n = 60, N = 2)R: 139.7±20.9 (n = 60, N = 2)(2-tailed Student’s *t*-test, p<0.01)	I: min. = 327.2±22.9 (n = 30)max. = 531.4±219.3 (n = 30)R: 416.5±87.2 (n = 60, N = 2)	I: 0.2±0.1(n = 60, N = 2) R: 0.3±0.1(n = 60, N = 2) (2-tailed Student’s *t*-test, p<0.001)
**Buzzing signals**	514.2±216.0 (n = 11, N = 2)	–	min. = 5.1±1.1(n = 7) max. = 9.8±1.4 (n = 4)

n = the number of signals analysed, N = the number of individuals analysed, where N is not specified then N = 1. Data are shown as mean ± SD when differences in parameter values between individuals were not significant and as minimal and maximal values when differences in parameter values between individuals were significant. For percussion signals we indicate the statistical test used to compare parameters of signals emitted as independent sequences (I) and parameters of signals emitted as a response to other vibrational emissions (R).

### Characteristics of Tremulatory, Percussion and Buzzing Signals

Males and females produced tremulatory signals **(** **Figures 1** **,** **2** **;** **Table 1** **)** vigorously shaking the body with legs firmly standing on the plant substrate. Pulses were produced with irregular repetition rate. Frequency spectrum shows a broad peak below 200 Hz and another peak around 260 Hz at 30 dB lower amplitude **(** **Figure 1** **, TR)**. Tremulatory signals recorded on the plant stem have a characteristic high frequency onset with a tail of prolonged low frequency vibration **(** **Figure 2** **)** and have been recorded on a plant in the calling phase of mating behaviour. A searching mate emitted these signals in the absence or presence of vibrational signals produced by signalling bugs on the neighbouring plant. We did not record this type of signals when bugs touched each other or refused to copulate as typical for investigated predatory bugs [16].

Percussion signals **(** **Figures 1** **,** **2** **)** also were emitted by males and females, and were recorded during searching phase of a single insect on the plant or together with the exchange of male and female signals produced by abdomen vibration. Percussion signals were emitted as sequences of regularly repeated pulses as a response to other vibrational signals produced by the mate or were emitted as independent sequences. Percussion pulse duration was statistically significantly different when signals were emitted as a response to other vibrational signals compared to when they were emitted as independent sequences. Statistical differences between signals emitted as a response to other vibrational signals and those emitted in independent sequences were also recorded for signal velocity **(** **Table 1** **)**. Frequency spectrum of percussion signals **(** **Figure 1** **, PER)** shows similar general characteristics as the one of tremulatory signals: a broad peak below 200 Hz and a peak of lower amplitude around 260 Hz.

In two males we observed vigorous vibration of lifted wings that produced high amplitude plant vibrations **(** **Figures 1** **,** **2** **;** **Table 1** **)**. Buzzing signals were repeated randomly with repetition time between 5 and 12 s. Their spectra show basic characteristics of abdomen vibration signals. Buzzing signals are characterized by narrow dominant frequency peak at 117.1±10.3 Hz (n = 11, N = 2) and regularly repeated higher harmonic peaks up to 1000 Hz with peak amplitude not falling more than 60 dB below that of the dominant frequency **(** **Figure 1** **, BZ)**. The behavioural context of buzzing signal production is not clear and shows no connection to vibrational emissions of other bugs.

### Airborne Inter-plant Communication

**Figure 3:**
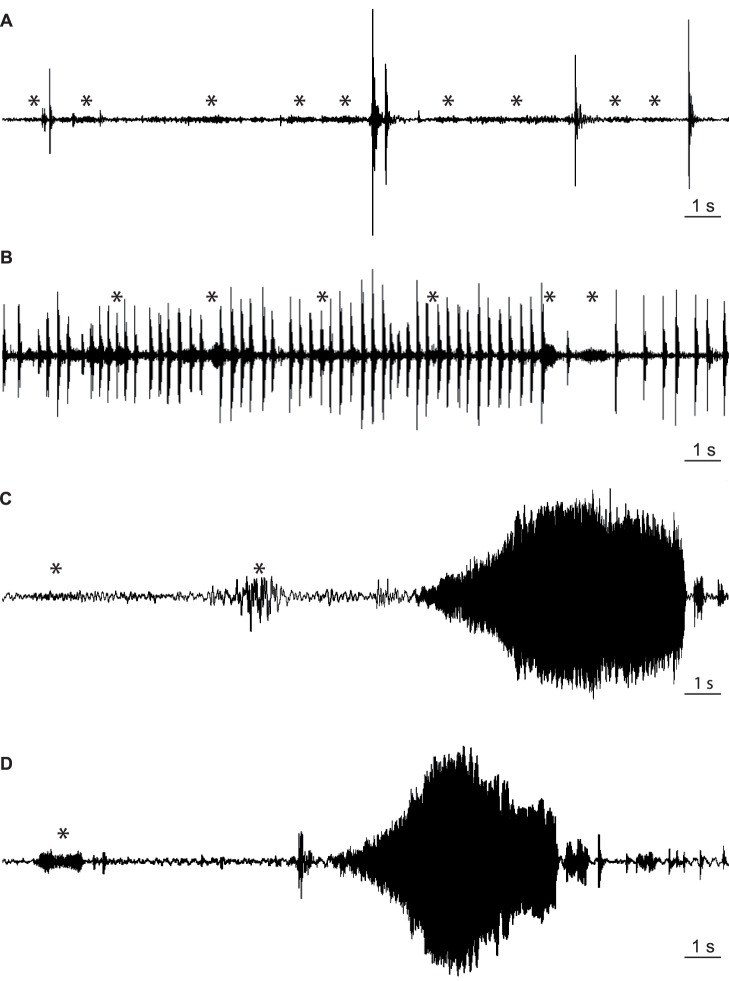
Vibrational responses to abdominal vibration produced signals on a neighbouring plant. Tremulatory (A), percussion (B) and abdomen vibration produced signals (C and D) emitted by a male as a response to abdomen vibrational signals (asterisks) produced on a neighbouring plant. Plants were in contact with the tips of the two closest leaves (A, B and C) or were separated by a 3–7 cm wide gap of air (D).

A single male or female placed on a plant exhibited different activities when the plant was not vibrated (control) or when vibrated with signals emitted naturally on a neighbouring plant in contact or non-contact conditions **(** **Table 2** **)**. In control conditions no searching and/or directional movement to the closest leaf of the neighbouring plant could be observed. Moreover, significantly (Fisher’s exact test for count data, p<0.05) less bugs stayed on the plant until the end of the test and lower number of tremulatory signals was emitted. On the other hand, normal communication between individuals on different plants with signals produced by abdomen vibration, tremulation and percussion **(** **Figure 3** **)** was recorded on a plant when in contact with another plant on which *E. heros* couples were singing: single males and females searched the source of vibration and moved directionally to the leaf in contact with neighbouring plant eventually moving on it over leaves in contact with their tips.

**Table 2:** Comparison of animal activity on a non-vibrated and on a vibrated plant.

	CONTROL (N = 20)	TEST (N = 29)
**staying on the plant**	15	28 (p<0.05)*
**emitting vibrational signals**	13	23 (p = 0.3314, NS)
**searching behaviour**	0	11
**reaching the point on the leaf closest to the neighbouring plant**	0	8

Number of individual animals exhibiting different activity levels on the plant in control and in test conditions. Fisher’s exact test for count data was used to compare between control and test conditions the number of individual animals staying on the plant and the number of individual animals emitting vibrational signals. N represents the number of individuals.

When plants were separated by 3 to 7 cm air gap significantly (Fisher’s exact test for count data, p<0.05) less bugs left the plant before the end of the test and higher percent of them searched and reached the point on the leaf that was closest to the leaf of the neighbouring plant. The number of tremulatory signals emitted per minute was 10.5±7.7 (n = 34, N = 4), which was higher compared to 4.4±1.9 (n = 16, N = 2) tremulatory signals emitted per minute in control conditions, where there were no animals on the neighbouring plant. The main difference observed between conditions when plants were in contact and conditions with no mechanical contact, was in the type of the song emitted: in non-contact conditions (and in control) single males or females produced mainly tremulatory signals while in contact conditions they predominantly emitted signals produced by abdomen vibration.

**Figure 4:**
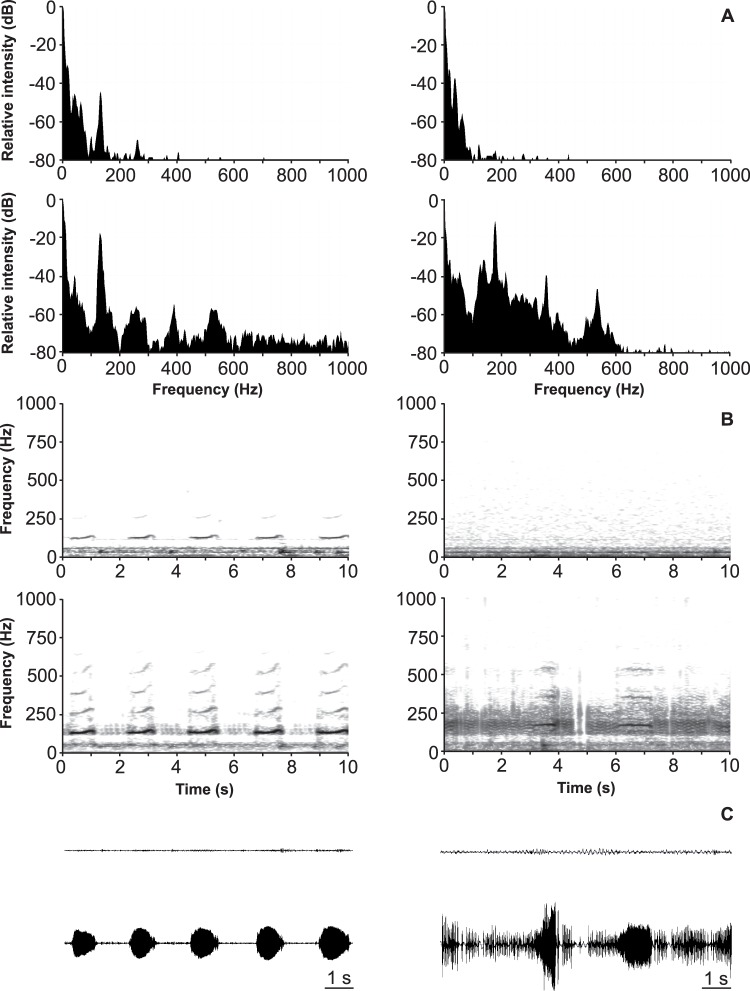
Spectral characteristics of vibrational signals before and after transmission through air. Frequency spectra (A) (one pulse), sonograms (B) (10 s sequence) and oscillograms (C) (10 s sequence) of abdomen vibration produced signals, recorded simultaneously from the plant where insects were singing (lower traces) and from the neighbouring plant (upper traces). The two plants were separated by an approximately 3 (left) or 6 (right) cm air gap.

Frequency spectra of naturally emitted *E. heros* vibrational signals differ when recorded simultaneously on two plants separated by an air gap of approximately 3 or 6 cm **(** **Figure 4** **)**. Transmission over the air from one to another plant shows low-pass filtering with increasing cut-off of frequencies above 300 Hz with increasing distance between both plants. The velocity difference between simultaneously recorded signals on two plants separated by an air gap of approximately 3 cm varied in three bugs between 22.5±7.8 (n = 11) and 29.3±5.6 (n = 11) dB for abdomen vibration produced signals and between 24.7±6.7 (n = 7) and 32.2±5.7 (n = 11) dB for tremulatory signals emitted naturally. At approximately 6 cm air distance the velocity difference was measured only for one animal. For simultaneously recorded signals it was 26.6±7.6 (n = 11) dB for abdomen vibrations and 34.3±2.6 (n = 11) dB for tremulatory signals.

## Discussion

This study first demonstrated that stink bugs of the subfamily Pentatominae emit vibrational signals produced by tremulation, percussion and buzzing in addition to the previously described abdominal vibrational signals [24]. The emission of high amplitude tremulatory and buzzing signals with low species specificity is an advantage in long range inter-plant communication, but on the other hand increases the risk of erroneous decisions in mate recognition and of attracting predators.

The role of tremulatory signals has been described in the Neotropical katydid *Docidocercus gigliotosi* Griffini (Orthoptera: Tettigoniidae) in a cost-benefit analysis of public and private communication [25], and in meadow katydids during courtship as an important parameter for female preference for large male vibrations [26]. Tremulatory signals have been first described in Pentatomidae in the predatory stink bug species *Podisus maculiventris* [16] and *Picromerus bidens* [18]. Males produced sequences of regularly repeated pulse trains composed of a tremulatory signal followed by an abdominal vibration produced pulse [16]. Tremulatory signals were recorded on a loudspeaker in male and female *P. bidens* when touching each other, when repelling a mate trying to copulate and as a response to female song signals [18].

Results of the present study on the pentatominae stink bug *E. heros* on a plant indicate that tremulatory signals have an important function in the calling phase of mating behaviour. A combination of long range oriented tremulatory signal with the shorter range species-specific signal produced by abdomen vibration has not been previously observed. Percussion signals have been recorded in *P. maculiventris* in connection with abdominal vibrations in the pause between pulses or as longer sequences following them [16]. We have recorded tremulatory signals in *E. heros* males and females either when they were alone or singing in a duet on a plant, as well as when responding to airborne signals. The role of *E. heros’* percussion and buzzing signals in communication needs further behavioural studies.

Results of the present study showed inter-plant communication with airborne vibrational signals in the stink bug *E. heros* and opened a new modality of information exchange in Heteroptera plant-dwelling insects on the same and on different plants. Two sensory systems are able to detect airborne signals involved in *E. heros* communication. The first includes leg vibrational receptors detecting vibrations of the plant substrate that were induced by airborne component of substrate-borne signals emitted on another plant. Simultaneous recording of naturally emitted *E. heros* vibrations on one plant and vibrations induced by its airborne component on the other shows 20–40 dB velocity decay at the air gap of 3 to 7 cm. Velocity values of plant recorded *E. heros* signals range between 1 and 10 mm/s for tremulatory and buzzing signals, between 0.1 and 1 mm/s for abdomen vibration signals and between 0.01 and 0.1 mm/s for percussion signals. At velocity decay of vibrational signals transmitted from one plant to the other through an air gap, as measured in our experimental conditions, we can expect that only values of percussion signals fall below the threshold of the low and high frequency leg vibrational receptor organs described in the related pentatomid species *N. viridula* [27].

Highly sensitive hair sensilla situated on the insect’s body represent the second sensory input for direct detection of airborne sound. Amputation of antennae did not decrease female *S. titanus* leafhopper response to conspecific airborne male calling song signals [5], indicating that sensilla on other parts of the body may be involved. Stink bug *N. viridula* has trichobothria at the edge of abdomen [28] and high sensitivity of these sensilla has been determined in the Heteroptera species *Pyrrhocoris apterus* [29]. Barth and Höller [30] demonstrated that spider’s trichobothria of 0.5 mm length detect airflow produced by a fly at a horizontal distance of 30 cm. Spectra of airflow signals produced by a fly show low frequency characteristics similar to those of *E. heros* tremulatory and buzzing signals, and their frequency rapidly decreases below 100 Hz at distances above 15 cm. Direct measurements of air particle movements around the tremulating, buzzing or abdomen vibrating stink bug have not been conducted yet but we can hypothesize that their airborne component is high enough to involve this sensory input both at the close and long range airborne sound communication.

Frequency decay with distance of airborne signals as shown in a spider-fly model [30] was confirmed in our study when comparing spectra of simultaneously recorded signals induced naturally on one and by airborne signals on another plant. We can conclude that the use of low frequency and narrow band vibrational signals had an advantage in evolution of communication in plant-dwelling insects not only because of their tuning with mechanical properties of herbaceous plants [31], [23] but also because of their better transmission through the air. Frequency increase of airborne signals with increasing distance as documented in *S. titanus* [5] was not measured in *E. heros*. This difference may be caused by different frequency properties of vibrational signals and/or by different way of test plant vibration.

Responsiveness of small plant-dwelling insects like leafhoppers and pentatominae stink bugs to vibrations transmitted through air does not coincide with the hypothesis that insects’ small body size prevents efficient emission of low-frequency airborne sound [3], [4]. Low-frequency and narrow-band vibrational signals induce vibrations of leaves of dimensions much larger compared to the insect. Moreover, it was shown that the amplitude of naturally emitted vibrational signals of *Nezara viridula* is significantly higher on leaves than on the stem [32]. If the amplitude of leaf induced low-frequency vibrations is high enough, we can assume that the airborne component of this substrate-borne vibrational signal may induce vibrations of the leaf with which it is not in physical contact. With the amplitude not falling below the sensitivity threshold of insect’s vibrational receptors, these signals can be detected by the animal on the adjacent leaf.

In conclusion, we showed that pentatominae stink bugs *E. heros* in addition to species- and sex-specific vibrational signals produced by abdomen vibrations communicate also with substrate-borne vibrational signals produced by other mechanisms, and that information exchange can take place between individuals on physically separated substrates. Our behavioural observations together with measurements of frequency and velocity decay of vibrational signals when transmitted through air suggest that high-amplitude low-frequency tremulatory signals propagate particularly well in the signaling medium/context examined.

## Materials and Methods

### Experimental Animals and Plants

**Figure 5:**
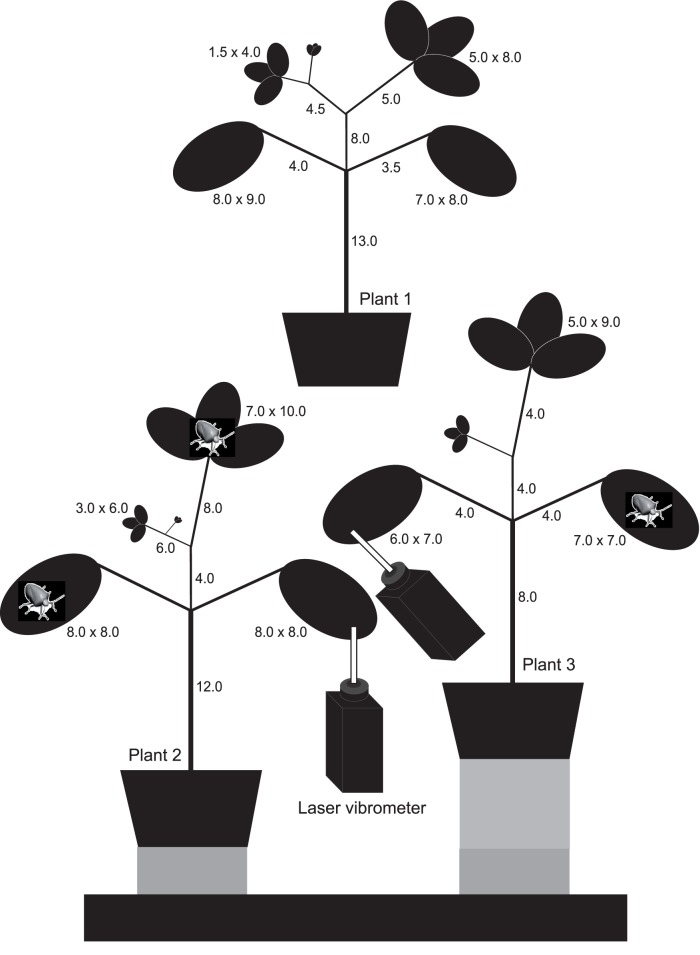
Architecture of bean plants (*Phaseolus vulgaris* L.) and experimental setup. Structure and dimensions of bean plants used, and experimental setup in airborne inter-plant communication experiments (below). See text for detailed description of experimental procedures.

Males and females from a colony reared *Euschistus heros* (Heteroptera: Pentatomidae), following the methodologies described by Borges et al. [33], were separated two days after the final moult and reared in the environmental room (26±1°C, 60±10% RH, 14∶10 h LD photoperiod under fluorescent lights of 40W) in plastic cages of 26 cm height and 22 cm diameter on raw peanut seeds (*Arachis hypogaea* L.), green beans (*Phaseolus vulgaris* L.) and sunflower seeds (*Helianthus annuus* L.). Vibrational signals were recorded between 9.00 and 17.00 h under laboratory conditions (26±1°C, 65±10% RH, laboratory light) in a sound insulated room at Embrapa Genetic Resources and Biotechnology in Brasilia (Brazil). Virgin males and females were used in the experiments 15–25 days after the final moult to ensure their sexual maturity [34], [35]. *Phaseolus vulgaris* L. test plants, grown in sterilized soil in plastic pots of 20 cm height and 15 cm width, were placed on a shock-proved table to reduce environmental sound and vibration noise **(** **Figure 5** **)**.

### Registration of Vibrational Signals

The experimental setup for registration of *E. heros* vibrational signals consisted of a single *P. vulgaris* plant potted in a plastic pot and placed on a rubber base to exclude unwanted substrate borne vibrations. The test started by placing a male on a leaf of a plant and waiting for 3 minutes to record spontaneous vibration emissions. After three minutes we put a female on the same plant and their behaviour was observed for 15 minutes. Vibrations were registered by a laser vibrometer (PDV-100, Polytec, Waldbronn, Germany) with the beam oriented perpendicularly at the surface of the plant’s stem at a distance 14–16 cm from the point where the bugs was singing. To get better laser beam reflection a small piece of reflecting tape (> 1 mm^2^ surface) was glued on the recording point on the plant. The analysis of signal characteristic was conducted by the Sound Forge software, version 6.0 (Sonic Foundry Inc., Madison, WI), the signals were digitized and stored via a sound card (24-bit, 96-kHz, 100-dB signal-to-noise ratio, Sound Blaster Extigy, Creative Laboratories Inc., Milpitas, CA) on a computer by the aid of the Cool Edit Pro software version 2.0 (Adobe Systems Inc., San Jose, CA).

Pulses defined as unitary homogenous parcels of vibrations of finite duration [36] were described by their duration (time between signal onset and its end determined by the amplitude of the cycle that decreased to the level of the noise range), repetition time (time between onsets of the two sequential signals) and by frequency spectra and sonagrams. Frequency spectra and sonograms have been constructed by the Sound Forge software (32768 FFT, 99% FFT overlap, Blackman-Harris smoothing window, slices displayed 1 and 9000 sonogram resolution). Velocity as vector quantity specifying the time rate of displacement was measured with the laser vibrometer at 5 mm/s/V sensitivity at the maximum amplitude of the pulse.

### Airborne Inter-plant Communication

The experimental setup in airborne inter-plant communication experiments consisted of two *P. vulgaris* plants placed in pots on separate rubber bases in order to exclude interplant transmission of substrate-borne vibrations **(** **Figure 5** **)**. Plants were either in contact or separated by the air gap, defined in our experiments as the distance between two most adjacent leaves of the two neighbouring plants.

Experiments were conducted at different gap distances, ranging from 3 to 11 cm in successive steps of approximately 2 cm. Initial gap widths were 3, 5, 7, 9 and 11 cm. Because of the leaf architecture and animals moving on the plats, the initial distance between the two most adjacent leaves changed significantly during the experiments. What is more, at gap distances up to 7 cm bugs’ responses did not differ, while at distances above 7 cm no response was observed. For this reasons, we did not distinguish in the text between individual gap widths but focused on the range of gap distances where animals’ responses were recorded.

Signals were recorded from the plant with a single bug using one laser vibrometer or simultaneously from both plants using two identical laser vibrometers. Recording points are shown in Figure 5.

The test started by placing a male on a leaf of one plant for 3 minutes to record spontaneous vibration emissions. After three minutes we put a female on the same plant, but on a different part of it so that the mates could not see each other, and when they started singing we placed a male or a female on the neighbouring plant. Vibrational signals were recorded from a plant with a single bug using one laser or simultaneously from both plants using two identical laser vibrometers.

A single male or a female on one plant and no animals on the neighbouring plant presented control conditions, and a single male or a female on one plant and a male-female pair of *E. heros* signalling on the neighbouring plant presented test conditions. In both cases the neighbouring plants were separated by a gap of air. Insects were observed during a 15 min interval.

The choice of aspects of insects’ behaviour that were recorded in control and test conditions stems from the fact that successful communication in the stink bug *E. heros* during mating involves emission of species- and sex-specific vibrational signals and directional movement of a male toward the singing female, known as searching behaviour. This behavioural pattern, common to some other pentatomidae species, is in nature triggered by the species-specific male pheromone with long-range action. This enables distant mates to meet on the same plant substrate where middle- and short-range substrate-borne vibrational communication can take place. If communication is successful, it leads to copulation.

In the experiment we recorded the number of animals staying on the plant throughout the experiment, the emission of vibrational signals, searching behaviour and directional movement towards the neighbouring plant. To check whether the number of tremulatory signals emitted is different between control and test conditions, which would indicate that these signals could be involved in *E. heros’*s vibrational communication, as was reported for some Asopinae species, we compared the tremulation rate between control and test conditions.

We hypothesised that substrate-borne vibrational signals could be transmitted over the gap to the neighbouring plant, which would result in inter-plant communication indicated by a higher number of animals exhibiting the recorded behaviour and by higher tremulation rate in test conditions compared to control conditions.

In order to test how transmission through air of substrate-borne vibrational signals affects their characteristics, we measured velocity and frequency changes of the signals by simultaneously recording vibrations from both neighbouring plants.

Our assumption was that after transmission through air substrate-borne vibrational signals would retain their low frequency characteristics, typical of Pentatomidae stink bugs’ vibrational signals, and that the amplitude of high-amplitude vibrational signals would not fall below the threshold of the bugs’ sensory organs.

Data are represented as means ± SD (n- the number of signals analysed, N- the number of individuals) when there were no statistically significant differences between groups of values, and as minimum and maximum values when statistically significant differences existed between groups of values.

Assuming normal distribution and homogeneity of variances 2-tailed Student’s *t*-test and one-way ANOVA were used. Fisher’s exact test for count data was used to compare animal activity in inter-plant communication experiments.
